# Comparative Action of Quinia, Cinchona and Cinchonidia

**Published:** 1877-10

**Authors:** 


### The Comparative Action of Quinia, Cinchona and Cinchonidia.

MM. Laborde and Dupuis recently made a report on the physiological action of these drugs to the Societé de Biologie. These experiments were made upon dogs, and the results are published in *La Tribune Médicale* for the seventeenth of June last. Since cinchonidia is now being so largely used in place of quinia, these experiments will prove interesting to the readers of the *Monthly.*

The amounts of the different drugs used were about 15 grains of quinine and about 12 grains of each of the other articles—cinchonine and cinchonidine.

The dog who received a hypodermic injection of 15 grains of quinine, at the end of half an hour was in a stupefied condition, and there was a very marked loss of general sensibility, and soon afterwards there was complete anæsthesia. The second dog, who had received the hypodermic injection of 12 grains of cinchonine, after certain prodromic symptoms, which consisted in restlessness and a wild expression, and also some spasmodic twitches, such as are produced by electricity, suddenly " uttered a cry," followed by two or three other sounds of a similar character, and then fell to the ground and had a generalized tonic convulsion, attended by foaming at the mouth and the involuntary discharge of urine. Upon this followed a period marked by clonic convulsions and stertorous breathing. M. Laborde says there could not have been a more distinctly marked attack of epilepsy. These attacks passed off in a short time, to be repeated at intervals for some hours, and it was noteworthy that they were readily brought on by peripheral excitation.

The third dog, who had received about 12 grains of cinchonidine by hypodermic injection, after a prodromic period marked by trembling very similar to that of paralysis agitans, was taken with an epileptic attack similar in all respects to the one we have just described, except that it was a less violent.

The result of these experiments show the propriety of caution when giving large doses of cinchona and cinchonida in place of quinine.— *Virginia Medical Monthly.*

